# Supplementary material for: Two incursions, two viruses: emergence of a second novel Shamonda virus clade, Germany, 2026
Source: Euro Surveill. 2026 Oct 1;31(39):2600756. doi: 10.2807/1560-7917.ES.2026.31.39.2600756 (PMC13633466; doi:10.2807/1560-7917.ES.2026.31.39.2600756)
Supplement: Supplementary file 1 [file 26-00756_BEER_Supplement.pdf]

Supplemental Material

for

Two incursions, two viruses: Emergence of a second novel Shamonda virus clade in Central Europe, 2026

This supplementary material is hosted by *Eurosurveillance* as supporting information alongside the article [Two incursions, two viruses: Emergence of a second novel Shamonda virus clade in Central Europe, 2026], on behalf of the authors, who remain responsible for the accuracy and appropriateness of the content. The same standards for ethics, copyright, attributions and permissions as for the article apply. Supplements are not edited by *Eurosurveillance* and the journal is not responsible for the maintenance of any links or email addresses provided therein.

Supplemental Material and Methods

Metagenomic sequencing

RNA was extracted from plasma samples using the RNAdvance Tissue Kit (Beckman Coulter) on a KingFisher Duo Prime platform (Thermo Fisher Scientific). For sequencing, the RNA was further processed as previously described [1]. In addition, after size selection, the libraries were amplified using AccuPrime Pfx DNA Polymerase and subsequently size selected again. Thereafter, libraries were quantified as described [1]. Pooled libraries were loaded onto Ion 530 chips using an Ion Chef instrument and sequenced on an Ion S5 XL platform in 400-bp mode. The obtained datasets were assembled with Newbler (v3.0; Roche/454) and for nine samples a segmented viral genome with similarity to orthobunyaviruses was obtained. The resulting complete coding sequences are available from the INSDC (International Nucleotide Sequence Database Collaboration, http://www.insdc.org) databases under project no. PRJEB125115.

Phylogenetic analysis

Phylogenetic analyses were performed separately for all three genome segments. Initially, for a broader phylogenetic classification appropriate reference sequences (RefSeq) of the genus *Orthobunyavirus* were retrieved from GenBank and viruses belonging to the Simbu, Bunyamwera, California, Leanyer and Sedlec serogroups were selected. The amino acid sequences were aligned using MUSCLE (v5.1; [2]) and Maximum-likelihood trees were inferred with IQ-TREE (v3.1.3; [3]) using ModelFinder (-m MFP; [4]) to select the best-fitting amino acid substitution model. Tree searches were performed using exhaustive nearest-neighbor interchange optimization (-allnni). Branch support was assessed using 10,000 ultrafast bootstrap replicates [5] and 10,000 SH-aLRT replicates. Bootstrap trees were additionally optimized using nearest-neighbor interchange (-bnni).

For the analyses of nucleotide sequences, reference sequences from the species *Orthobunyavirus schmallenbergense* (Douglas virus, Sathuperi virus, Schmallenberg virus and Shamonda virus) were retrieved from GenBank. Subsequently, the coding sequences (CDSs) were first identified using "Find ORF" function implemented in Geneious Prime (v2025.1.3) and subsequently extracted. The extracted CDSs were aligned using MUSCLE (v5.1) and Maximum-likelihood phylogenetic trees were inferred with IQ-TREE (v3.1.3; [3]) using a partitioned analysis [6]. In detail, the alignments were partitioned by codon position (first, second, and third positions) to account for the possible different evolutionary constraints and substitution rates among codon positions. ModelFinder was used to determine the best-fitting substitution model for each partition and to optimize the partitioning scheme (-m MFP+MERGE; [4]). Branch support statistics and NNI optimization was done as described above.

Supplemental Figures

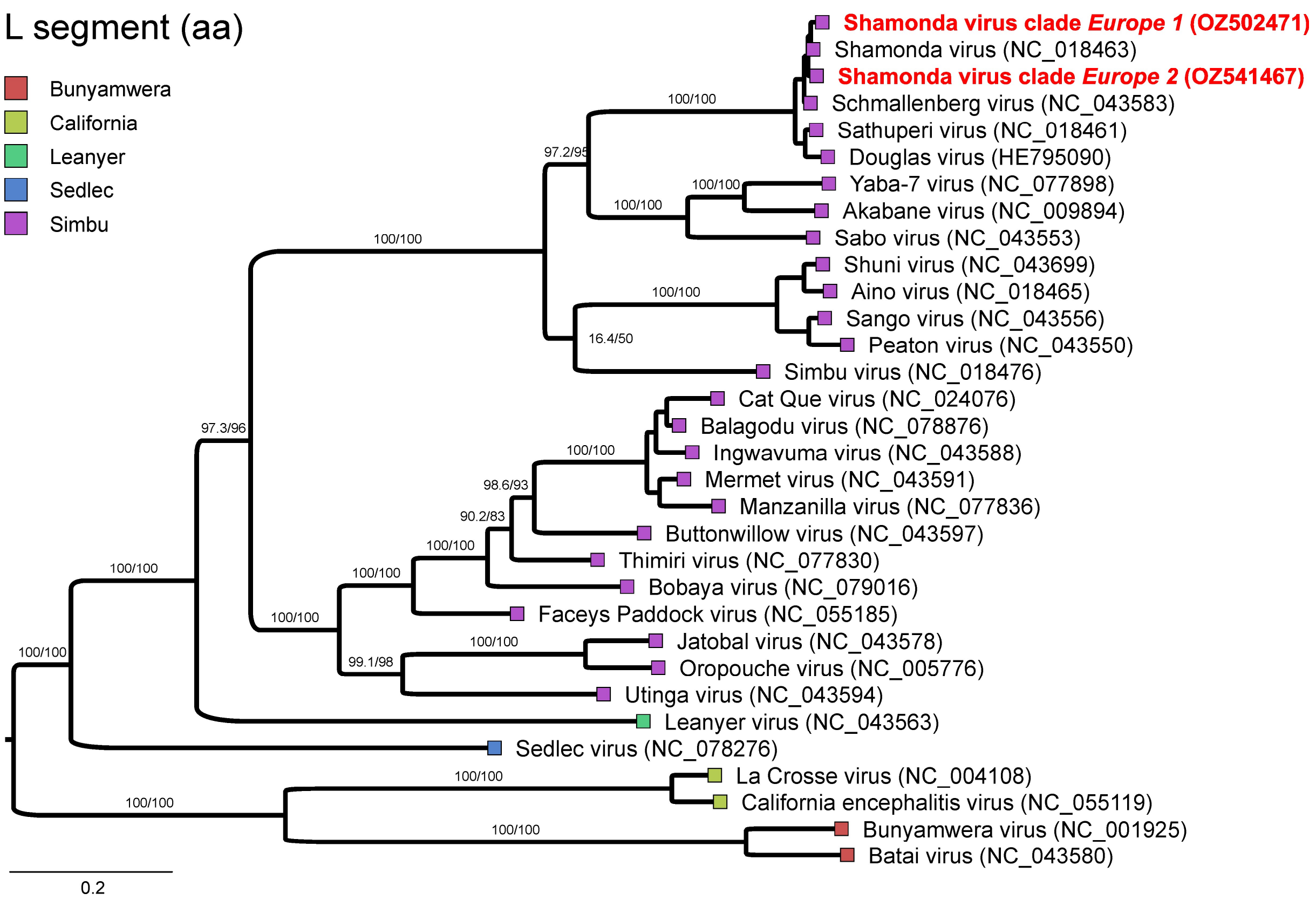

Supplemental Figure S1: Maximum-likelihood phylogenetic analysis of the L segment of selected orthobunyaviruses. Coding sequences of the L protein were extracted, translated, and aligned using MUSCLE (v5.1). Phylogenetic inference was performed with IQ-TREE (v3.1.3), with the optimal amino acid substitution model selected using ModelFinder (-m MFP). The maximum-likelihood tree search was performed using exhaustive nearest-neighbor interchange optimization (-allnni). Branch support was assessed using 10,000 SH-aLRT and 10,000 ultrafast bootstrap replicates, with additional nearest-neighbour interchange optimization of bootstrap trees (-bnni). Viruses are coloured according to their serogroup. The two newly detected Shamonda virus clades, Europe 1 and Europe 2, are highlighted in bold red text. Branch support is shown for relevant nodes as SH-aLRT support (%) / ultrafast bootstrap support (%).

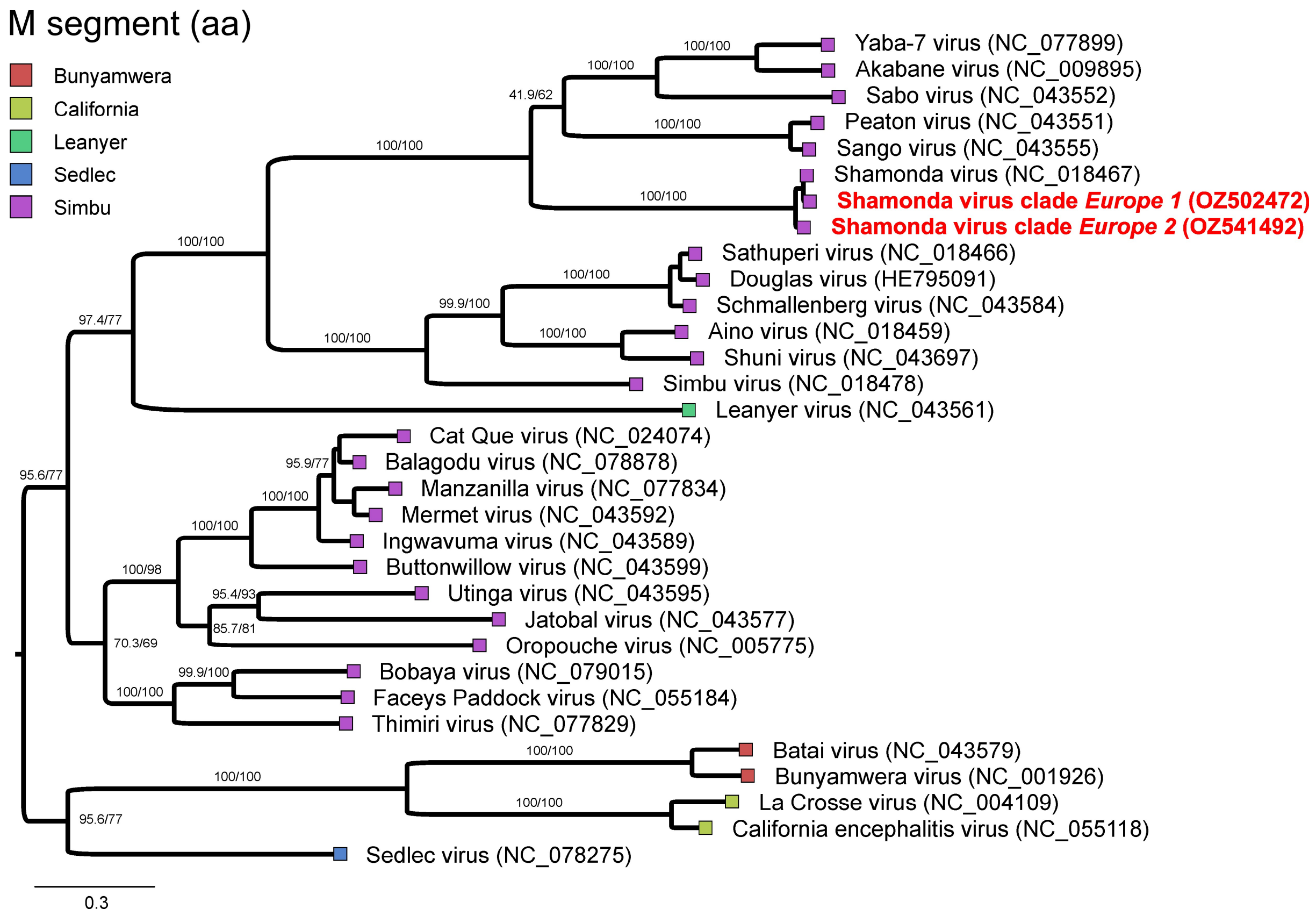

Supplemental Figure S2: Maximum-likelihood phylogenetic analysis of the M segment of selected orthobunyaviruses. Coding sequences of the glycoprotein precursor were extracted, translated, and aligned using MUSCLE (v5.1). Phylogenetic inference was performed with IQ-TREE (v3.1.3), with the optimal amino acid substitution model selected using ModelFinder (-m MFP). The maximum-likelihood tree search was performed using exhaustive nearest-neighbor interchange optimization (-allnni). Branch support was assessed using 10,000 SH-aLRT and 10,000 ultrafast bootstrap replicates, with additional nearest-neighbour interchange optimization of bootstrap trees (-bnni). Viruses are coloured according to their serogroup. The two newly detected Shamonda virus clades, Europe 1 and Europe 2, are highlighted in bold red text. Branch support is shown for relevant nodes as SH-aLRT support (%) / ultrafast bootstrap support (%).

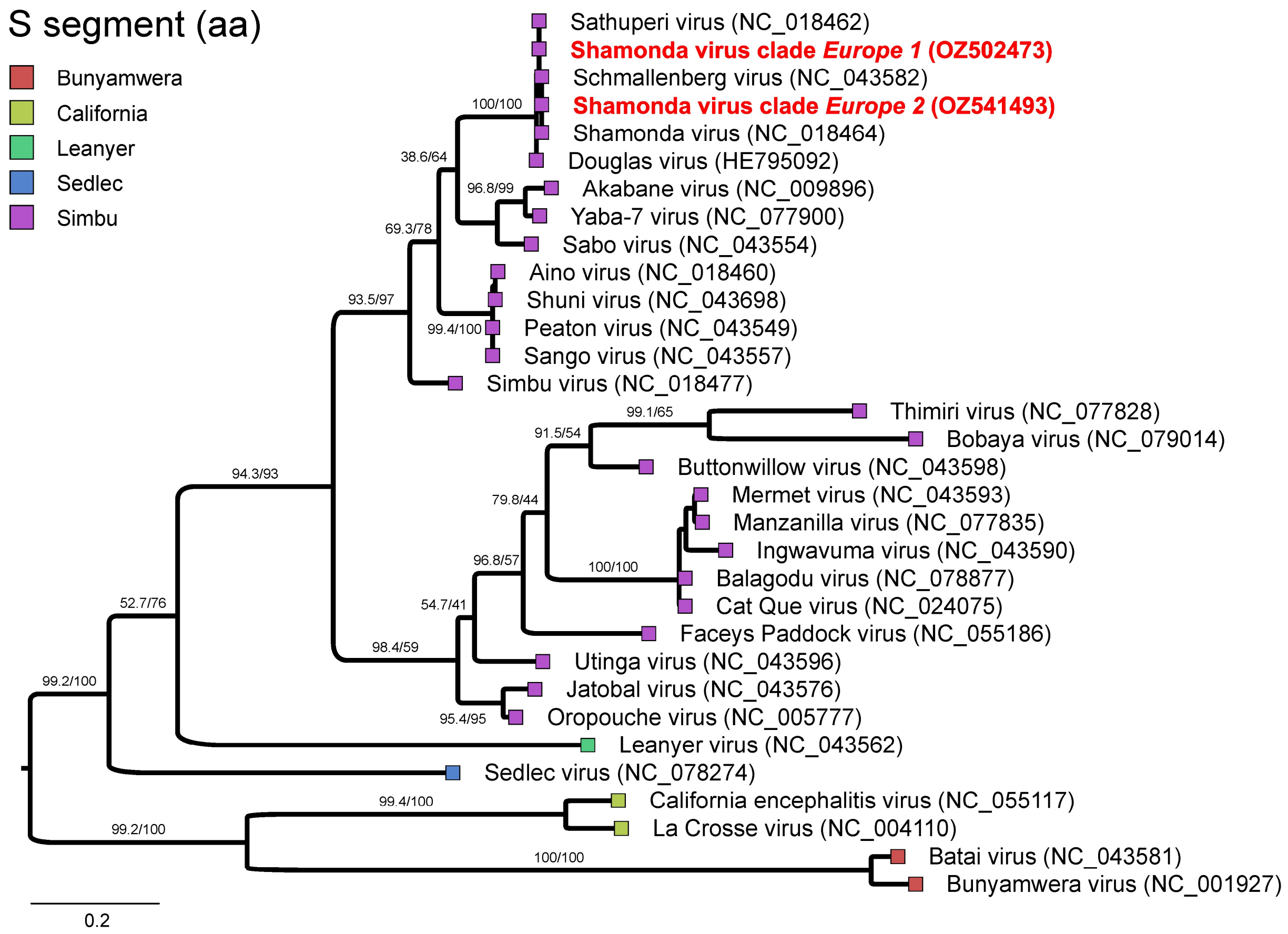

Supplemental Figure S3: Maximum-likelihood phylogenetic analysis of the S segment of selected orthobunyaviruses. Coding sequences of the nucleocapsid protein were extracted, translated, and aligned using MUSCLE (v5.1). Phylogenetic inference was performed with IQ-TREE (v3.1.3), with the optimal amino acid substitution model selected using ModelFinder (-m MFP). The maximum-likelihood tree search was performed using exhaustive nearest-neighbor interchange optimization (-allnni). Branch support was assessed using 10,000 SH-aLRT and 10,000 ultrafast bootstrap replicates, with additional nearest-neighbour interchange optimization of bootstrap trees (-bnni). Viruses are coloured according to their serogroup. The two newly detected Shamonda virus clades, Europe 1 and Europe 2, are highlighted in bold red text. Branch support is shown for relevant nodes as SH-aLRT support (%) / ultrafast bootstrap support (%).

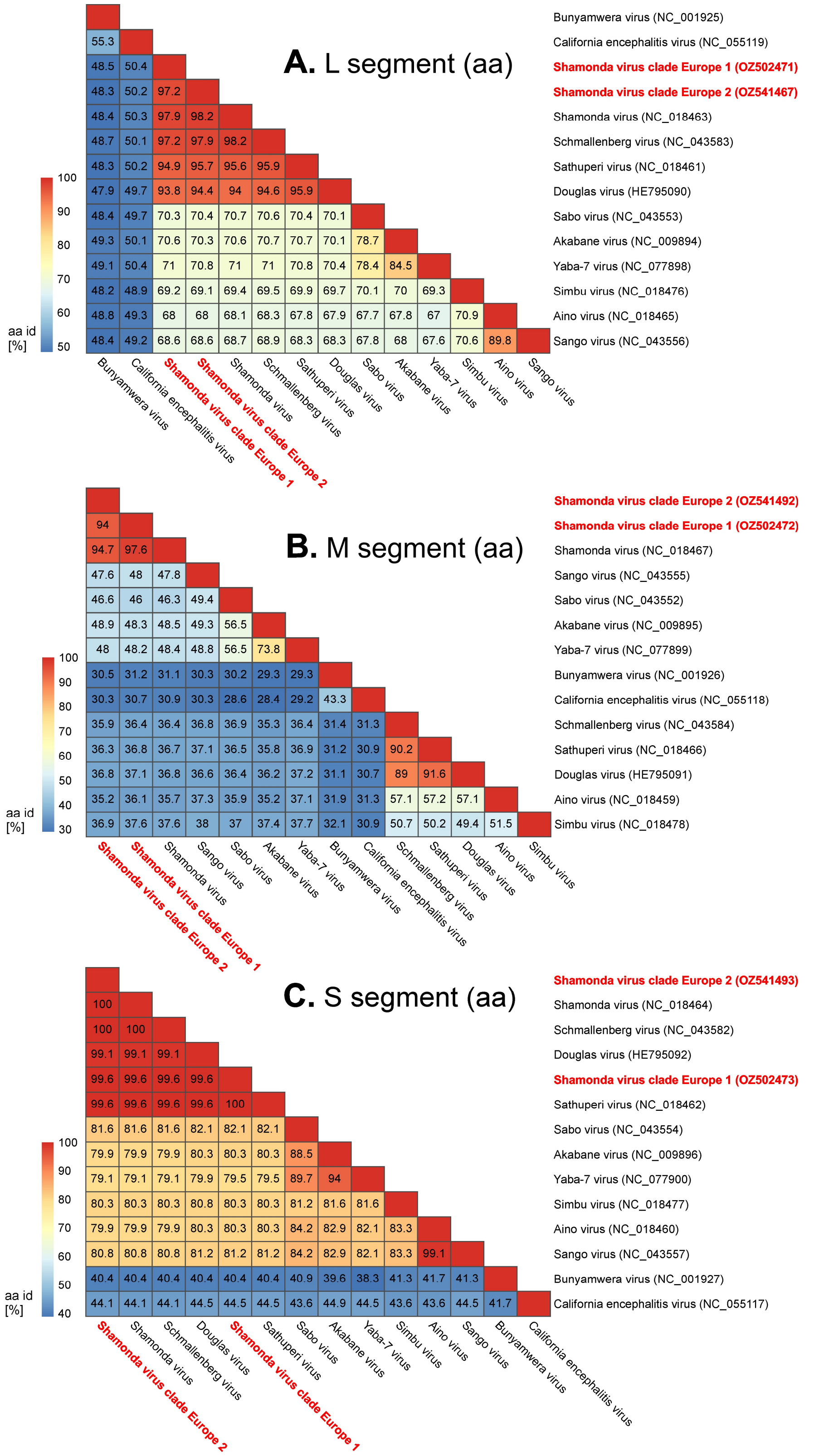

Supplemental Figure S4: Pairwise amino acid identity of selected orthobunyaviruses. Coding sequences of the L protein (**A.**), the glycoprotein precursor (**B.**) and the nucleocapsid protein (**C.**) were extracted, translated, and aligned using MUSCLE (v5.1). The pairwise amino acid identity was deduced and plotted. The two newly detected Shamonda virus clades, Europe 1 and Europe 2, are highlighted in bold red text.

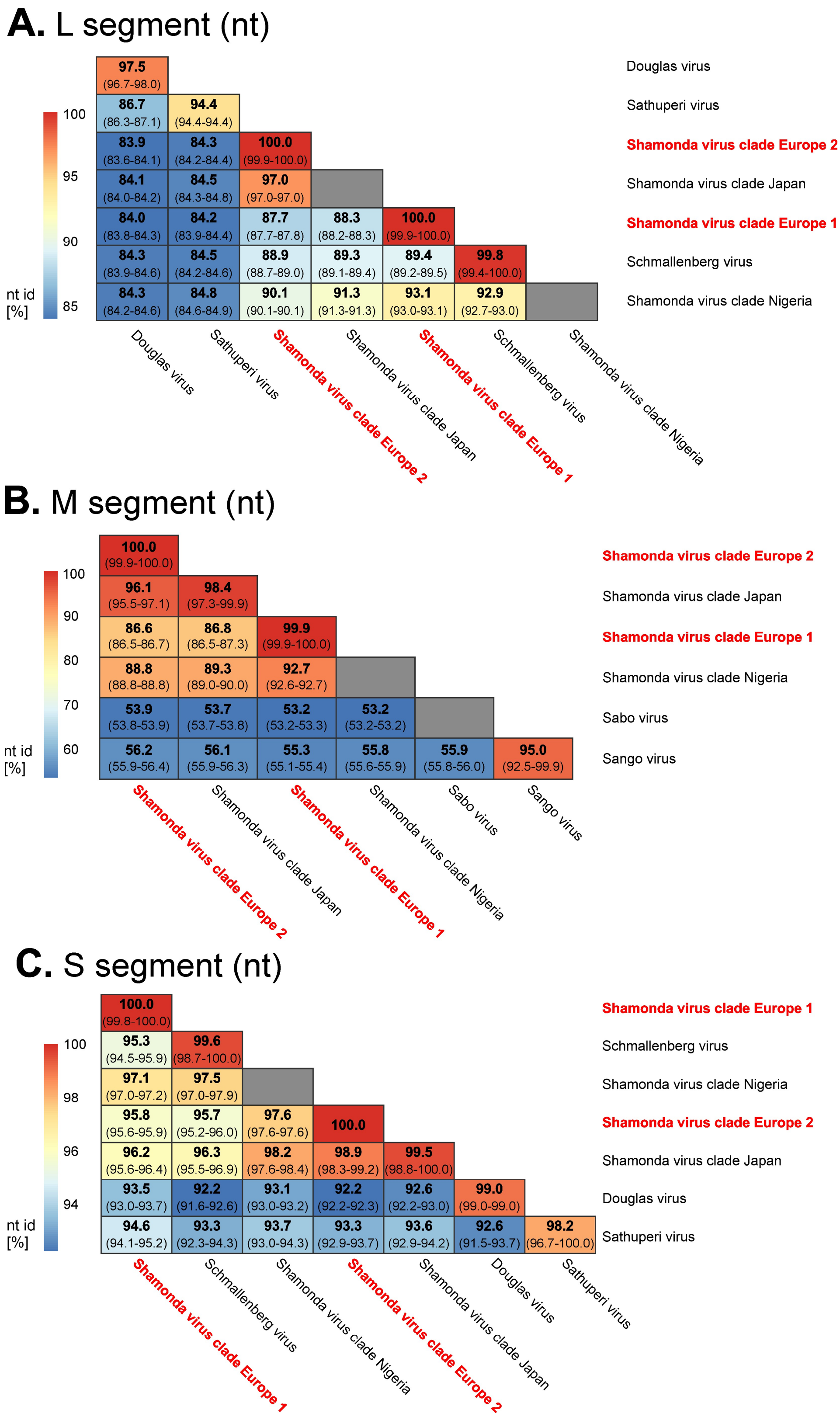

Supplemental Figure S5: Pairwise nucleotide identity of selected orthobunyaviruses. Coding sequences of the L protein (**A.**), the glycoprotein precursor (**B.**) and nucleocapsid protein (**C.**) were extracted and aligned using MUSCLE (v5.1). Pairwise nucleotide identities were calculated for all included sequences and summarized for each virus pair as the mean identity, with the corresponding minimum–maximum range shown below. The two newly detected Shamonda virus clades, Europe 1 and Europe 2, are highlighted in bold red text.

Supplemental Table

Supplemental Table S1: Data on samples sent to the German National Reference Laboratory for Schmallenberg virus from animals suspected of having a Shamonda virus infection. Origin, sample material, date of sample receipt and RT-qPCR results are given for samples that tested positive by a pan-Simbuvirus RT-qPCR. Samples that were investigated by metagenomic sequencing are marked by an asterisk. BW - Baden-Württemberg, BY - Bavaria, HE - Hesse, NI - Lower Saxony, NW - North Rhine-Westphalia, RP - Rhineland-Palatinate

Sample ID	Origin	Sample material	Date	Pan-Simbu RT-qPCR	S-segment-based SBV RT-qPCR	L-segment-based SBV RT-qPCR
2026BVD09542	Germany, NW	serum	18-08-2026	27.7 / positive	28.7 / positive	negative
2026BVD09543	Germany, NW	serum	18-08-2026	26.7 / positive	28.5 / positive	negative
2026BVD09544	Germany, NW	serum	18-08-2026	24.9 / positive	26.6 / positive	negative
2026BVD09545	Germany, NW	serum	18-08-2026	28.8 / positive	30.5 / positive	negative
2026BVD09546	Germany, NW	serum	18-08-2026	32.3 / positive	32.8 / positive	negative
2026BVD09547	Germany, NW	serum	18-08-2026	33.9 / positive	34.2 / positive	negative
2026BVD09548	Germany, NW	serum	18-08-2026	36.2 / positive	39.7 / positive	negative
2026BVD09699*	Germany, NW	plasma	19-08-2026	23.7 / positive	27.1 / positive	negative
2026BVD09701*	Germany, NW	plasma	19-08-2026	22.8 / positive	26.3 / positive	negative
2026BVD09702	Germany, NW	plasma	19-08-2026	24.9 / positive	28.1 / positive	negative
2026BVD09703	Germany, NW	plasma	19-08-2026	23.9 / positive	26.5 / positive	negative
2026BVD09711	Germany, NW	plasma	19-08-2026	33.3 / positive	35.1 / positive	negative
2026BVD09712*	Germany, NW	plasma	19-08-2026	23.3 / positive	26.4 / positive	negative
2026BVD09714	Germany, NW	plasma	19-08-2026	24.1 / positive	26.6 / positive	negative
2026BVD09715	Germany, NW	plasma	19-08-2026	25.4 / positive	28.2 / positive	negative
2026BVD09716	Germany, NW	plasma	19-08-2026	26.2 / positive	28.0 / positive	negative
2026BVD09717	Germany, NW	plasma	19-08-2026	27.8 / positive	29.6 / positive	negative
2026BVD09722	Germany, NW	plasma	19-08-2026	24.3 / positive	27.2 / positive	negative
2026BVD09724	Germany, NW	plasma	19-08-2026	34.8 / positive	35.2 / positive	negative
2026BVD09725	Germany, NW	plasma	19-08-2026	25.3 / positive	28.5 / positive	negative
2026BVD09726	Germany, NW	plasma	19-08-2026	24.7 / positive	27.4 / positive	negative
2026BVD09727	Germany, NW	plasma	19-08-2026	35.2 / positive	39.6 / positive	negative
2026BVD09728	Germany, NW	plasma	19-08-2026	30.2 / positive	31.7 / positive	negative
2026BVD09729	Germany, NW	serum	19-08-2026	23.7 / positive	26.1 / positive	negative

Sample ID	Origin	Sample material	Date	Pan-Simbu RT-qPCR	S-segment-based SBV RT-qPCR	L-segment-based SBV RT-qPCR
2026BVD09753	Germany, NW	EDTA blood	20-08-2026	23.9 / positive	26.3 / positive	negative
2026BVD09754	Germany, NW	EDTA blood	20-08-2026	29.8 / positive	28.9 / positive	negative
2026BVD09755	Germany, NW	EDTA blood	20-08-2026	26.4 / positive	26.1 / positive	negative
2026BVD09756	Germany, NW	EDTA blood	20-08-2026	25.8 / positive	29.2 / positive	negative
2026BVD09757	Germany, NW	EDTA blood	20-08-2026	26.9 / positive	33.2 / positive	negative
2026BVD09758	Germany, NW	serum	20-08-2026	26.7 / positive	28.4 / positive	negative
2026BVD09759	Germany, NW	serum	20-08-2026	24.2 / positive	26.2 / positive	negative
2026BVD09760	Germany, NW	serum	20-08-2026	24.3 / positive	26.5 / positive	negative
2026BVD09761	Germany, NW	serum	20-08-2026	26.5 / positive	27.6 / positive	negative
2026BVD09768	Germany, NW	EDTA blood	20-08-2026	24.5 / positive	26.2 / positive	negative
2026BVD09769	Germany, NW	EDTA blood	20-08-2026	23.3 / positive	25.3 / positive	negative
2026BVD09770	Germany, NW	EDTA blood	20-08-2026	24.1 / positive	26.5 / positive	negative
2026BVD09771*	Germany, NW	EDTA blood	20-08-2026	21.7 / positive	24.6 / positive	negative
2026BVD09772	Germany, NW	EDTA blood	20-08-2026	25.7 / positive	27.3 / positive	negative
2026BVD09774*	Germany, NW	EDTA blood	20-08-2026	21.6 / positive	24.1 / positive	negative
2026BVD09775	Germany, NW	EDTA blood	20-08-2026	30.8 / positive	32.9 / positive	negative
2026BVD09776	Germany, NW	EDTA blood	20-08-2026	29.5 / positive	30.8 / positive	negative
2026BVD09780	Germany, NW	EDTA blood	20-08-2026	24.5 / positive	27.3 / positive	negative
2026BVD09549	Germany, NI	EDTA blood	18-08-2026	30.6 / positive	30.3 / positive	negative
2026BVD09818	Germany, NI	EDTA blood	20-08-2026	25.0 / positive	26.3 / positive	negative
2026BVD09819	Germany, NI	EDTA blood	20-08-2026	20.3 / positive	23.9 / positive	negative
2026BVD09820	Germany, NI	EDTA blood	20-08-2026	25.2 / positive	26.1 / positive	negative
2026BVD09821*	Germany, NI	EDTA blood	20-08-2026	23.0 / positive	24.8 / positive	negative
2026BVD09822	Germany, NI	EDTA blood	20-08-2026	24.4 / positive	26.6 / positive	negative
2026BVD09823	Germany, NI	EDTA blood	20-08-2026	32.5 / positive	33.3 / positive	negative
2026BVD09824	Germany, NI	EDTA blood	20-08-2026	25.6 / positive	27.3 / positive	negative
2026BVD09825	Germany, NI	EDTA blood	20-08-2026	35.3 / positive	35.5 / positive	negative
2026BVD09826	Germany, NI	EDTA blood	20-08-2026	36.3 / positive	37.7 / positive	negative
2026BVD09828	Germany, NI	EDTA blood	20-08-2026	29.3 / positive	29.4 / positive	negative
2026BVD09829*	Germany, NI	EDTA blood	20-08-2026	23.3 / positive	25.2 / positive	negative
2026BVD09830	Germany, NI	EDTA blood	20-08-2026	27.5 / positive	28.9 / positive	negative
2026BVD09831	Germany, NI	EDTA blood	20-08-2026	33.2 / positive	34.0 / positive	negative
2026BVD09210	Germany, HE	EDTA blood	13-08-2026	22.1 / positive	negative	22.7 / positive
2026BVD09784	Germany, HE	EDTA blood	20-08-2026	26.0 / positive	27.6 / positive	negative

Sample ID	Origin	Sample material	Date	Pan-Simbu RT-qPCR	S-segment-based SBV RT-qPCR	L-segment-based SBV RT-qPCR
2026BVD09785	Germany, HE	EDTA blood	20-08-2026	31.1 / positive	31.9 / positive	negative
2026BVD09786	Germany, HE	EDTA blood	20-08-2026	21.2 / positive	negative	23.8 / positive
2026BVD09788	Germany, HE	EDTA blood	20-08-2026	24.7 / positive	negative	27.2 / positive
2026BVD09790	Germany, HE	EDTA blood	20-08-2026	26.6 / positive	negative	27.8 / positive
2026BVD09791	Germany, HE	EDTA blood	20-08-2026	21.6 / positive	negative	23.9 / positive
2026BVD09792	Germany, HE	EDTA blood	20-08-2026	26.4 / positive	negative	28.2 / positive
2026BVD09793*	Germany, HE	EDTA blood	20-08-2026	22.4 / positive	24.6 / positive	negative
2026BVD09794	Germany, HE	EDTA blood	20-08-2026	29.6 / positive	30.4 / positive	negative
2026BVD09795	Germany, HE	EDTA blood	20-08-2026	29.0 / positive	30.5 / positive	negative
2026BVD09796	Germany, HE	EDTA blood	20-08-2026	37.8 / positive	36.5 / positive	negative
2026BVD09797*	Germany, HE	EDTA blood	20-08-2026	24.4 / positive	26.3 / positive	negative
2026BVD09798	Germany, HE	serum	20-08-2026	20.8 / positive	negative	23.0 / positive
2026BVD09801	Germany, HE	EDTA blood	20-08-2026	20.9 / positive	negative	23.8 / positive
2026BVD09802	Germany, HE	EDTA blood	20-08-2026	25.5 / positive	negative	27.4 / positive
2026BVD09803	Germany, HE	EDTA blood	20-08-2026	27.7 / positive	29.0 / positive	negative
2026BVD09804	Germany, HE	EDTA blood	20-08-2026	22.3 / positive	negative	24.7 / positive
2026BVD09805	Germany, HE	EDTA blood	20-08-2026	25.3 / positive	27.1 / positive	negative
2026BVD09806	Germany, HE	EDTA blood	20-08-2026	25.2 / positive	negative	28.2 / positive
2026BVD09809	Germany, HE	EDTA blood	20-08-2026	34.4 / positive	34.0 / positive	negative
2026BVD09731	Germany, BW	EDTA blood	19-08-2026	32.7 / positive	negative	32.9 / positive
2026BVD09732	Germany, BW	EDTA blood	19-08-2026	22.2 / positive	negative	23.9 / positive
2026BVD09733	Germany, BW	serum	19-08-2026	29.2 / positive	negative	30.3 / positive
2026BVD09734	Germany, BW	serum	19-08-2026	23.0 / positive	negative	24.9 / positive
2026BVD09735	Germany, BW	EDTA blood	19-08-2026	21.1 / positive	negative	23.5 / positive
2026BVD09737	Germany, BW	EDTA blood	19-08-2026	22.1 / positive	negative	23.6 / positive
2026BVD09739	Germany, BW	serum	19-08-2026	22.2 / positive	negative	24.0 / positive
2026BVD09740	Germany, BW	EDTA blood	19-08-2026	21.5 / positive	negative	23.1 / positive
2026BVD09741	Germany, BW	EDTA blood	19-08-2026	24.8 / positive	negative	27.5 / positive
2026BVD09742	Germany, BW	serum	19-08-2026	20.2 / positive	negative	22.7 / positive
2026BVD09743	Germany, BW	serum	19-08-2026	23.1 / positive	negative	25.6 / positive
2026BVD09744	Germany, BW	EDTA blood	19-08-2026	20.5 / positive	negative	23.4 / positive
2026BVD09745	Germany, BW	EDTA blood	19-08-2026	19.3 / positive	negative	21.8 / positive
2026BVD09746	Germany, BW	serum	19-08-2026	22.4 / positive	negative	25.0 / positive
2026BVD09747	Germany, BW	serum	19-08-2026	18.5 / positive	negative	22.3 / positive

Sample ID	Origin	Sample material	Date	Pan-Simbu RT-qPCR	S-segment-based SBV RT-qPCR	L-segment-based SBV RT-qPCR
2026BVD09260	Germany, BY	serum	14-08-2026	19.6 / positive	negative	21.5 / positive
2026BVD09261	Germany, BY	serum	14-08-2026	24.9 / positive	negative	27.0 / positive
2026BVD09262	Germany, BY	serum	14-08-2026	37.2 / positive	negative	39.7 / positive
2026BVD09263	Germany, BY	serum	14-08-2026	33.0 / positive	negative	35.9 / positive
2026BVD09264	Germany, BY	serum	14-08-2026	34.4 / positive	negative	37.0 / positive
2026BVD09265	Germany, BY	serum	14-08-2026	19.8 / positive	negative	20.7 / positive
2026BVD09266	Germany, BY	serum	14-08-2026	24.1 / positive	negative	25.1 / positive
2026BVD09267	Germany, BY	serum	14-08-2026	35.3 / positive	negative	38.6 / positive
2026BVD09269	Germany, BY	serum	14-08-2026	25.1 / positive	negative	26.4 / positive
2026BVD09270	Germany, BY	serum	14-08-2026	22.3 / positive	negative	23.1 / positive
2026BVD07609	Germany, RP	EDTA blood	06-08-2026	26.4 / positive	negative	26.5 / positive
2026BVD07611	Germany, RP	EDTA blood	06-08-2026	24.6 / positive	negative	24.2 / positive
2026BVD07613	Germany, RP	EDTA blood	06-08-2026	25.1 / positive	negative	25.4 / positive
2026BVD07614	Germany, RP	EDTA blood	06-08-2026	23.7 / positive	negative	23.4 / positive
2026BVD07615	Germany, RP	EDTA blood	06-08-2026	33.2 / positive	negative	31.9 / positive
2026BVD07619	Germany, RP	EDTA blood	06-08-2026	29.6 / positive	negative	28.5 / positive
2026BVD08010	Germany, RP	EDTA blood	06-08-2026	22.4 / positive	negative	22.7 / positive
2026BVD08011	Germany, RP	EDTA blood	06-08-2026	24.4 / positive	negative	24.6 / positive
2026BVD08012	Germany, RP	EDTA blood	06-08-2026	23.8 / positive	negative	22.1 / positive
2026BVD08013	Germany, RP	EDTA blood	06-08-2026	24.3 / positive	negative	23.5 / positive
2026BVD08015	Germany, RP	EDTA blood	06-08-2026	23.3 / positive	negative	24.0 / positive
2026BVD08016	Germany, RP	EDTA blood	06-08-2026	26.1 / positive	negative	25.3 / positive
2026BVD08017	Germany, RP	EDTA blood	06-08-2026	28.0 / positive	negative	27.2 / positive
2026BVD08018	Germany, RP	EDTA blood	06-08-2026	23.0 / positive	negative	23.4 / positive
2026BVD08019	Germany, RP	EDTA blood	06-08-2026	25.0 / positive	negative	25.2 / positive
2026BVD08020	Germany, RP	EDTA blood	06-08-2026	25.3 / positive	negative	25.0 / positive
2026BVD08021	Germany, RP	EDTA blood	06-08-2026	24.1 / positive	negative	21.3 / positive
2026BVD08022	Germany, RP	EDTA blood	06-08-2026	25.0 / positive	negative	24.9 / positive
2026BVD08023	Germany, RP	EDTA blood	06-08-2026	26.6 / positive	negative	27.1 / positive
2026BVD08024	Germany, RP	EDTA blood	06-08-2026	34.4 / positive	negative	39.4 / positive

99

Supplemental References

1. Eschbaumer M, Staubach C, Pfaff F, et al. Buffaloed in Brandenburg: Germany's first Brush with Foot-and-Mouth Disease after four Decades of Freedom, **2026**.

2. Edgar RC. MUSCLE: multiple sequence alignment with high accuracy and high throughput. Nucleic Acids Res **2004**; 32:1792–7.

3. Wong TKF, Ly-Trong N, Ren H, et al. IQ-TREE 3: phylogenomic inference software using complex evolutionary models. Mol Biol Evol **2026**; 43.

4. Kalyaanamoorthy S, Minh BQ, Wong TKF, Haeseler A von, Jermiin LS. ModelFinder: fast model selection for accurate phylogenetic estimates. Nat Methods **2017**; 14:587–9.

5. Hoang DT, Chernomor O, Haeseler A von, Minh BQ, Le Vinh S. UFBoot2: Improving the Ultrafast Bootstrap Approximation. Mol Biol Evol **2018**; 35:518–22.

6. Chernomor O, Haeseler A von, Minh BQ. Terrace Aware Data Structure for Phylogenomic Inference from Supermatrices. Syst Biol **2016**; 65:997–1008.
